# Melanopsin-mediated amplification of cone signals in the human visual cortex

**DOI:** 10.1098/rspb.2023.2708

**Published:** 2024-05-29

**Authors:** Prakash Adhikari, Samir Uprety, Beatrix Feigl, Andrew J. Zele

**Affiliations:** ^1^ Centre for Vision and Eye Research, Queensland University of Technology (QUT), Brisbane, Queensland 4059, Australia; ^2^ School of Biomedical Sciences, Queensland University of Technology (QUT), Brisbane, Queensland 4059, Australia; ^3^ Queensland Eye Institute, Brisbane, Queensland 4101, Australia

**Keywords:** melanopsin, cones, vision, visual evoked potentials, electroretinogram

## Abstract

The ambient daylight variation is coded by melanopsin photoreceptors and their luxotonic activity increases towards midday when colour temperatures are cooler, and irradiances are higher. Although melanopsin and cone photoresponses can be mediated via separate pathways, the connectivity of melanopsin cells across all levels of the retina enables them to modify cone signals. The downstream effects of melanopsin-cone interactions on human vision are however, incompletely understood. Here, we determined how the change in daytime melanopsin activation affects the human cone pathway signals in the visual cortex. A 5-primary silent-substitution method was developed to evaluate the dependence of cone-mediated signals on melanopsin activation by spectrally tuning the lights and stabilizing the rhodopsin activation under a constant cone photometric luminance. The retinal (white noise electroretinogram) and cortical responses (visual evoked potential) were simultaneously recorded with the photoreceptor-directed lights in 10 observers. By increasing the melanopsin activation, a reverse response pattern was observed with cone signals being supressed in the retina by 27% (*p* = 0.03) and subsequently amplified by 16% (*p* = 0.01) as they reach the cortex. We infer that melanopsin activity can amplify cone signals at sites distal to retinal bipolar cells to cause a decrease in the psychophysical Weber fraction for cone vision.

## 1. Introduction

Between dawn and dusk, the melanopsin-expressing intrinsically photosensitive retinal ganglion cells (ipRGC), rod and three cone photoreceptor classes are active in humans. During these daylight hours, melanopsin activation increases towards midday when colour temperatures are cooler, and illuminations are higher. Through ipRGC projections to the visual cortex via the dLGN [1,2], preferential stimulation of melanopsin evokes haemodynamic responses in area V1 [3] and conscious visual perception [3–9]. To provide a unified visual percept during continual changes in the spectral power distribution of the environmental light, the visual system is therefore tasked with merging signals from the melanopsin and cone pathways having distinct spectral, spatial and temporal contrast responses. It is the convergence of cone and melanopsin signals that changes the behaviour of retinal circuits and potentially, at downstream post-retinal sites to affect visual functions. These downstream processes are not however well understood and are investigated here.

With higher levels of melanopsin excitation, the photopic cone-directed electroretinogram (ERG), as an index of the function of outer retinal cells, shows a suppression of the b-wave amplitude that is near-synchronous with the melanopsin activation [10–12]. Such inhibitory retinal interactions are thought to be mediated via retrograde signals between the ipRGCs and the outer retina [13,14]. Conversely, cone-mediated visual contrast sensitivity can increase with higher levels of melanopsin excitation [5,15,16], indicating that the suppressed retinal signals are amplified, with the site yet to be defined. To identify the cortical contribution to this interaction, we explore whether melanopsin activity can differentially modulate retinal and cortical signals to set cone-mediated visual sensitivity.

Most of our knowledge on how melanopsin shapes cone-mediated human processes is from ERG [10,17] and psychophysical studies [15,16,18,19] implementing sophisticated photoreceptor silent substitution protocols, with some approaches leaving the rod activation level uncontrolled. The significance of leaving rod excitations unrestrained is that melanopsin-directed stimulations inadvertently introduce concomitant rod intrusions due to overlapping melanopsin and rod spectral responses. In photopic lighting, mammalian rods can escape saturation [20–23] and interact with both cone [24] and melanopsin signals [16]. Given that the photopic rod-melanopsin [16] and cone-melanopsin interactions [5,15,16] can be of opposite polarity, the melanopsin enhancement of cone contrast sensitivity may be of lower magnitude [15] or completely nulled depending on the level of rod intrusion if it is left uncontrolled [18]. We therefore apply a custom-developed 5-primary spectral tuning method to silence rhodopsin while varying the melanopsin activation level. A full-field white noise electrophysiological technique simultaneously probes cone-directed retinal and cortical visual evoked responses (VEPs) at a constant photopic luminance. With this approach, we predict that the change in daylight melanopsin activity can differentially modify the cone-directed responses at early (outer retinal) and later (post-bipolar cell/cortical) sites within the intact, human visual system.

## 2. Material and methods

### (a) Participants and ethics statement

All experimental protocols were approved and carried out in accordance with the Queensland University of Technology (QUT) Human Research Ethics Committee approval (no. 1700000699) and followed the tenets of the Declaration of Helsinki; written informed consent was obtained by the authors from all participants. All 10 participants (3 females, 7 males; 29–47 years) had visual acuity of 0.0 logMAR (6/6) or better, age-normal spatial contrast sensitivity (Spatial Contrast Vision Chart) [25], trichromatic colour vision (Ishihara pseudoisochromatic plates and L'anthony Desaturated D-15 Test), no ocular diseases as confirmed with ophthalmoscopy, optical coherence topography (RS-3000 OCT RetinaScan Advance, Nidek Co., Tokyo, Japan), fundus photography (Canon Non Mydriatic Retinal Camera, CR-DGi, Canon Inc., Tokyo, Japan) and intraocular pressure measurement (less than 21 mmHg) (Icare ic100; Icare Finland Oy, Vantaa, Finland), and no systemic disease.

### (b) Apparatus

A custom-built 5-primary monocular Ganzfeld apparatus was developed to spectrally tune the full-field retinal illumination with independent control of all five photoreceptor excitations using the method of silent substitution [26,27]. A gamut suitable for complete photoreceptor control [28] was generated with five narrowband LED and interference filter combinations (Thorlabs, Newton, NJ, USA) with peak wavelengths (full width at half maximum) at 449 nm (15 nm) for the blue (B) primary, 497 nm (10 nm) for the cyan (C), 547 nm (25 nm) for the green (G), 593 nm (13 nm) for the amber (A) and 654 nm (13 nm) for the red (R). The 5-primary light outputs were modulated using an LED driver (TLC5940), microcontroller (Arduino Uno SMDR3, Model A000073), and custom designed software (Xcode 3.2.3, 64-bit, Apple, Inc., Cupertino, CA, USA) having 12-bit resolution and a high frequency limit of 488 Hz [5].

To assess the effect of melanopsin on cone pathway signals, the retinal wnERG and cortical wnVEP were simultaneously recorded using an Espion E2 system (Colordome; Diagnosys, Lowell, MA, USA) at a sampling rate of 1000 Hz and filtered between 0.3 and 300 Hz in accordance with the International Society for Clinical Electrophysiology of Vision (ISCEV) guidelines [29]. The protocol was triggered by an Apple MacPro QuadCore Intel computer, which controlled the trigger input to start the silent substitution protocol and ERG/VEP recording via a microcontroller (Arduino Uno SMD R3, Model A000073). System delays were accounted for during *post hoc* analyses.

### (c) Experimental design: photoreceptor spectral tuning protocols

For silent substitution, the physical light outputs and individual observer calibrations were performed in accordance with standardized protocols [27]. The photoreceptor-directed lights were specified initially with reference to the CIE 1964 10° standard observer cone spectral sensitivities, rhodopsin nomogram and melanopsin nomogram [30]. Because the spectral responses of rhodopsin and melanopsin are positively correlated [31], the maximum 59.6% melanopsin Weber contrast at the adapting chromaticity (1964 CIE*x*,*y* = 0.549, 0.410) would introduce a concomitant 43.0% rod Weber contrast intrusion if rhodopsin was not controlled in our silent substitution protocol [27,32]. Non-visual opsins identified in mammals, including humans, such as OPN3, OPN5 and a retinal G protein-coupled receptor, are not considered in this silent-substitution protocol due to a lack of direct evidence for any light-dependent role in human image-forming functions [27].

Individual observer calibrations were performed using heterochromatic flicker photometry (HFP) [33] to determine the scaling factors required to correct for individual deviations in pre-receptoral filtering and photoreceptor spectral sensitivity from the CIE standard observer functions [27]. The instrument gamut was maximized with an orange-appearing adaptation background (*l* = 0.752, *s* = 0.105 and *r* = 0.319; 1964 CIE *x* = 0.549, *y* = 0.410) that differed by 52.6% (Weber contrast) between a lower and higher melanopsin excitation (*i*_low_ = 0.19 and *i*_high_ = 0.29) designed to intentionally envelope a broad range of the total daytime variation in melanopsin activity [34]. We changed the background melanopsin excitation independently of the cone and rhodopsin excitations at a fixed photometric luminance (159.2 cd m^−2^). We could therefore specify the cone-mediated electrophysiological responses with reference to the circadian equivalent daytime variation in melanopsin excitation (biological efficacy). This daytime variation of melanopsin is non-linearly related to the correlated colour temperature [34] and for our experimental conditions, varies between a lower (1645K; delta *u*,*v* = 0.0029) and higher state (1955K; delta *u*,*v* = 0.0039). The lower state served as the control [4,18,35]. The cone-directed temporal white noise (TWN) stimuli [36,37] modulated the L-, M- and S-cone photoreceptor excitations (20% Michelson contrast) in the same phase to photoreceptor-directed (LMS) cone luminance noise without introducing a change in the rhodopsin and melanopsin excitations.

Our calculations show that the differential absorption of the primary lights by the open-field cones and the macular pigment [38] can introduce ≤ 0.1% undesired luminance (L+M) contrast and ≤ 0.4% chromatic (+L-M) contrast errors in both the low and high melanopsin adapting backgrounds, and ≤ 0.2% L+M contrast and ≤ 0.4% +L-M contrast errors in the cone-directed white noise stimulus. Penumbral cones in the shadow of the retinal vasculature [7,39] might present ≤ 0.3% L+M contrast and ≤ 0.6% +L-M contrast with the adapting backgrounds, and ≤ 0.3% L+M contrast and 0.8% +L-M contrast for the cone-directed stimulus. It is important to emphasize that we did not generate a melanopsin-directed stimulus; we implemented cone-directed stimuli under steady-state adaptation that had either a lower or higher melanopsin excitation. This means that the imperfections in the cone photoreceptor isolation will be similar magnitude between the two background states and simply add to the cone stimulus contrast, without confounding the interpretation of the effect of melanopsin on cone signals. As such, these measured contrast imperfections are smaller than can be detected in an ERG recording [10,37,40–43], and at such levels, their magnitude is too low to modify cone-mediated visual thresholds [19]. Accordingly, none of our observers reported the appearance of Maxwell's spot [44] or a Purkinje tree [39], with the orangish appearing background [44,45], and with the balanced rod activity across the central and peripheral visual field [46].

### (d) Temporal white noise electrophysiology procedures

The electrodes were set-up according to the ISCEV protocol for ERG [47] and VEP recordings [29]. For the wnERG, an active fibre electrode (Diagnosys, Lowell, MA, USA) was placed across the lower conjunctiva. The ground (forehead) and reference (temple) gold (Ag/AgCl) cup electrodes (Diagnosys, Lowell, MA, USA) were filled with conductive gel (Aquasonic; Parker Laboratories, Fairfield, NJ, USA) and pressed firmly to adhere to the skin after the areas scrubbed with alcohol wipes and abrasive gel (Nuprep; D. O. Weaver & Co., Aurora, CO, USA). For the wnVEP, the gold cup scalp electrodes (Diagnosys, Lowell, MA, USA) were placed according to the international 10/20 system [29], positioned relative to the bony landmark on the skull between the anterior-posterior midline of nasion and inion. The active electrode was placed on the midline sagittal plane of the occipital scalp over the visual cortex (Oz); the reference electrode was placed over the frontal lobe (*Fz*), and a common ground electrode for both ERG and VEP recording was positioned at central vertex of the skull (*C_z_*). Electrode impedance was always below 5 k*Ω*.

A TWN stimulation paradigm [10,37,48,49] was used to electrophysiologically probe signals measured under conditions of retinal equilibrium with spectral tuning. Each 1 s TWN stimulus epoch contained 1024 photoreceptor excitations evenly distributed in the 0 to 64 Hz frequency range within a Gaussian distribution centred around the constant photopic adaptation level, with the phase varied randomly between 0° and 359°. The inverse fast Fourier transform of the TWN stimuli returns a constant amplitude spectrum in the 0 and 64 Hz temporal frequency range. To de-correlate the line frequency from the fundamental frequency of the stimulus and improve the signal to noise ratio of the averaged signal, each 1 s stimulus epoch was separated by a 1 ms blank interval set to the mean luminance and chromaticity [50]; the noise sequence was repeated to create 120 unique signal recordings during a total 120.12 s recording sequence. Each experimental condition was repeated twice so that all conditions had a minimum of 120 recordings per observer after artefact and blink removal. Electrophysiological signals were filtered to remove artefacts induced by blinks and large eye movements. The impulse response function (IRF) was derived by cross-correlating the TWN stimulus sequence with the filtered electrophysiological wnERG and wnVEP responses using custom-written Matlab software (R2022b; Mathworks, Natick, MA, USA). To maximize the SNR, the noise stimuli and ERG/VEP response were time locked using circular cross correlation [51,52]. This method offers an advantage over a flash ERG in that the entire recording sequence can be used for analysis in the form of frequency distributions.

### (e) General procedure

Stimuli were presented to the right eye in Newtonian view through the 3.81 cm Ganzfeld aperture. The pupil of the test eye was dilated (Tropicamide 1%; Bausch and Lomb, Australia; ≥ 8 mm diameter; 8000 Td). Following the electrode setup, participants underwent a 10 min dark adaptation. Prior to the presentation of the cone noise sequence, participants underwent preadaptation to either the low or high melanopsin adapting background to ensure recovery of melanopsin phototransduction from the onset of the background [35,53]. The order of presentation of the low and high melanopsin conditions was randomized. Between consecutive noise sequences, participants rested in the darkened laboratory. Recordings were performed at similar times to minimize the impact of circadian-dependent variation on melanopsin-mediated function [54].

### (f) Data analysis

**Figure 1.**
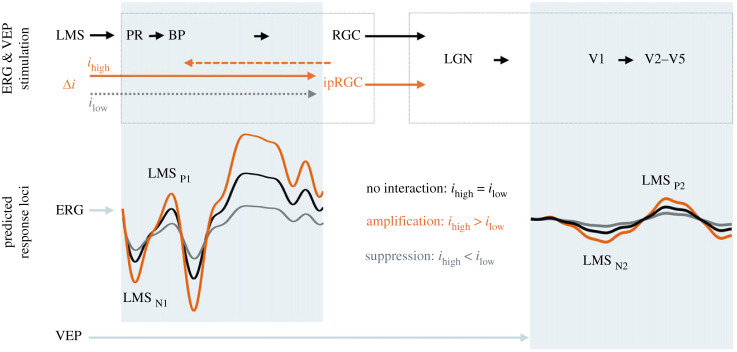
Predicted loci for the retinal (ERG waveforms) and cortical visual evoked potentials (VEP waveforms) recorded in response to cone-directed light stimulation (LMS) in the presence of lower (*i*_low_) or higher (*i*_high_) melanopsin excitation (*Δi*). The waveform predictions are with reference to the low melanopsin state, and the components (N1, P1, N2, P2) are aligned with their neural generators in the retina (ERG) and cortex (VEP). LMS cone pathway signalling is sequential from eye to brain (left to right, solid black arrows). Melanopsin ipRGCs feedforward (solid orange arrows, dotted grey arrow) and feedback (orange dashed arrow). PR, photoreceptors; BP, bipolar cells; RGC, retinal ganglion cells; ipRGC, intrinsically photosensitive RGCs; LGN, lateral geniculate nucleus; V1–5, visual areas 1–5.

All statistical analyses were conducted using GraphPad Prism (GraphPad Software, Inc., CA, USA). The data frequency distributions were estimated using the D'Agostino and Pearson omnibus normality test. The wnERG and wnVEP amplitudes and implicit times are reported as the mean ± s.e.m calculated across 10 observers. To detect the difference in the LMS-cone directed response between low melanopsin (*i*_low_) and high melanopsin (*i*_high_) excitations (figure 1), the wnERG and wnVEP amplitudes, implicit times and coefficient of variation (CoV) were compared using paired *t*-test (normally distributed data) or Wilcoxon test (non-normally distributed data) (95% confidence interval, *p* < 0.05). If there is no melanopsin-cone interaction, the LMS cone-directed ERG (N1P1) and VEP (N2P2) amplitudes will be equal between *i*_low_ and *i*_high_ (figure 1). If melanopsin enhances cone signals, the amplitudes will be higher with *i*_high_ than with *i*_low_. In the case of suppression, the amplitudes will be lower with *i*_high_ than with *i*_low_. Because the ERG and VEP signals originate from different neurones (figure 1), we calculated the ratio of the cone IRF amplitudes between the two melanopsin states (*i*_high_/*i*_low_) for each observer to determine how melanopsin adaptation affects the total relative change in cone signals reaching the cortex (VEP) from those originally generated in the retina (ERG). The initial VEP component (e.g. N2 in our waveforms) is most likely generated in the thalamocortical radiations [55] and striate cortex (Brodmann's area 17, V1), with the later component (e.g. P2 in our waveforms) arising from the extrastriate cortex (Brodmann's areas 18 (V2) and 19 (V3, V4 and V5); for review, see [56]), indicating that the N2P2 amplitude is dominated by cortical activity. We therefore use the N2P2 amplitude as a biomarker of the primary visual cortical response (figure 1). We predict that the amplitude ratio (*i*_high_/*i*_low_) will be unity if there is no interaction, less than unity with suppression, and greater than unity with amplification. The ratios were then compared between the ERG and VEP using paired *t*-test (or Wilcoxon). To identify the presence of time-dependent effect of melanopsin adaptation, linear regression models were fitted to the wnERG and wnVEP amplitudes or implicit times recorded over time (120 recording epochs per observer). The optimal bin width for the frequency distributions of the electrophysiological recordings were determined based on the sample size and standard deviation [57] and modelled using a hyperbolic secant function. To determine whether melanopsin alters the coding efficiency of cone signalling, intra-observer coefficient of variation (CoV = s.d/mean) across the 120 epochs was calculated as a measure of signal to noise ratio.

## 3. Results

**Figure 2.**
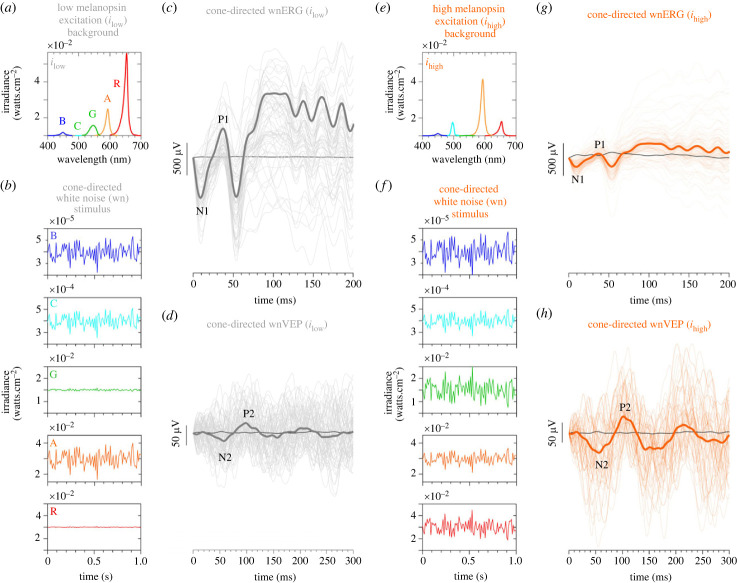
Retinal (wnERG) and cortical visual evoked potentials (wnVEP) recorded under conditions of photoreceptor silencing with lower (*i*_low_) or higher (*i*_high_) melanopsin excitations in the adapting background. (*a*) Spectral outputs of the 5-primary lights in the adapting background set to a melanopsin excitation having a low biological efficacy (*i*_low_). (*b*) Temporal irradiance modulation of each of the five primaries (presented separately in five panels) required to generate an LMS cone-directed temporal white noise (wn) probe under the low melanopsin background (*i*_low_), without changing rhodopsin. (*c*) Exemplar impulse response functions (IRF) of the cone-directed white noise electroretinogram (wnERG, grey lines) measured under low melanopsin excitation. Black line is the baseline recording (no white noise probe). (*d*) Exemplar visual cortical evoked potentials (wnVEP, grey lines) measured under low melanopsin excitation. (*e*) Spectral outputs of the primary lights in the adapting background to set the melanopsin excitation to a high biological efficacy (*i*_high_). (*f*) Temporal irradiance modulation of each of the five primary spectra to generate an LMS cone-directed white noise probe at a high melanopsin excitation (*i*_high_). (*g*) Exemplar cone-directed wnERG and (*h*) wnVEP measured under high melanopsin excitation (orange lines). The high and low melanopsin adaptation states (*a,e*) are equivalent for rods (i.e. rods silenced) and have a constant photometric luminance. To achieve the same LMS-cone temporal white noise stimulus contrast and amplitude pattern, and photometric luminance at both the low melanopsin (*b*) and high melanopsin (*f*) backgrounds, the 5-primaries have the same temporal noise amplitude patterns in the silent-substitution, but with different absolute irradiances to compensate for the different primary irradiance levels at the two backgrounds.

Our selection of a steady-state light adaptation protocol with five primaries (figure 2*a*: low melanopsin *i*_low_ background; figure 2*e*: high melanopsin *i*_high_ background) to completely specify all five photoreceptor excitations provides a clear separation of the effects of melanopsin and rhodopsin, with the high frequency, cone-directed white noise (wn) probe designed to limit intrusion from any abrupt temporal transient visual responses that occur with flash stimuli (figure 2*b*,*f*). Exemplar retinal wnERG (figure 2*c*,*g*) and cortical wnVEP (figure 2*d*,*h*) IRFs resemble the typical flash ERG and VEP, respectively; these were evident in all 10 observers.

**Figure 3.**
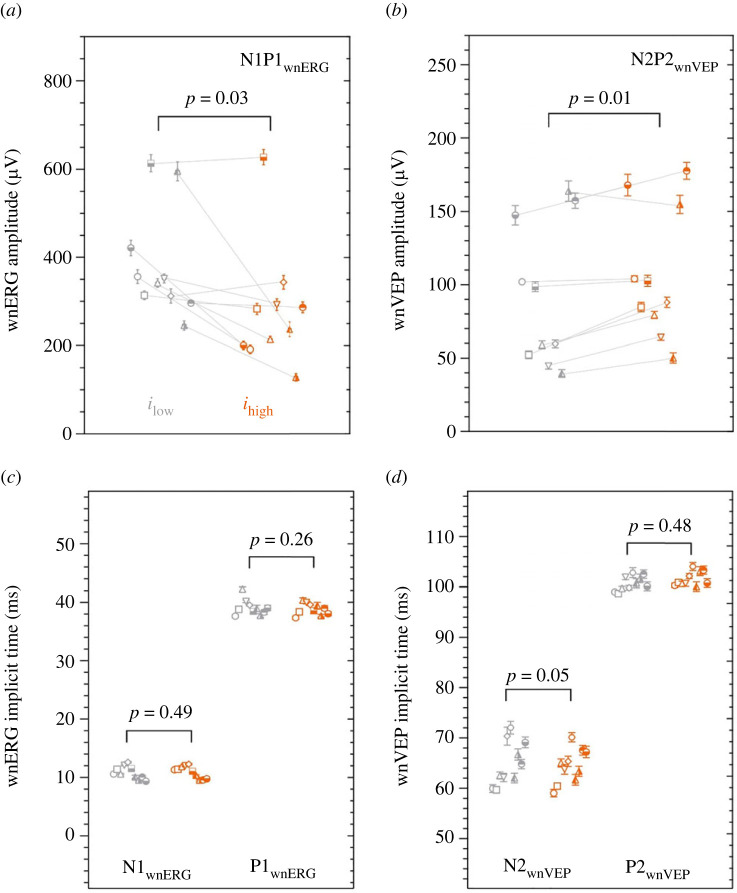
The cone-directed signals at the level of the retina (wnERG) and primary visual cortex (wnVEP). Each observer is represented by a unique symbol (mean ± s.e.m.); for each observer, the grey and orange symbols are connected by a grey line, indicating the transition from the low to high melanopsin excitation. (*a*) The N1P1 amplitudes of the cone-directed white noise electroretinogram (wnERG) measured under low melanopsin (grey symbols; *i*_low_) or high melanopsin excitation (orange symbols; *i*_high_) (*n* = 10). (*b*) N2P2 amplitudes for the cone-directed white noise visual evoked potential (wnVEP). (*c*) N1 and P1 implicit times for the wnERG. (*d*) N2 and P2 implicit times for the wnVEP.

In all 10 observers, the retinal IRF of the LMS-cone mediated wnERG (figure 3*c*) had an initial negative (N1) deflection at approximately11 ms (N1 implicit time: *i*_low_ = 10.80 ± 0.34 ms; *i*_high_ = 10.93 ± 0.33 ms; mean ± s.e.m.) followed by a positive (P1) deflection at approximately 39 ms (P1 implicit time: *i*_low_ = 39.11 ± 0.43 ms; *i*_high_ = 38.83 ± 0.32 ms). The cortical wnVEP (figure 3*d*) had a robust N2 component at approximately 65 ms (N2 implicit time: *i*_low_ = 64.96 ± 1.39 ms; *i*_high_ = 64.37 ± 1.08 ms) and P2 at ∼101 ms (P2 implicit time: *i*_low_ = 100.91 ± 0.45 ms; *i*_high_ = 101.63 ± 0.45 ms). The mean wnERG and VEP metrics from each observer were used to evaluate the dependence of cone function on melanopsin excitation, independent of changes in rhodopsin and the photometric luminance. The high melanopsin excitation caused a statistically significant 27.13% reduction (Wilcoxon *W* = −43.00, *p* = 0.03) in the mean cone-directed N1P1 wnERG amplitude (*i*_low_ = 384.84 ± 39.41 µV; *i*_high_ = 280.45 ± 43.30 µV; mean ± s.e.m.; figure 3*a*) and a significant 16.08% increase (*t*_9_ = 3.67, *p* = 0.01) in the cone-directed N2P2 wnVEP amplitude (*i*_low_ = 92.53 ± 15.41 µV; *i*_high_ = 107.21 ± 14.04 µV; figure 3*b*). All implicit times were invariant of the background melanopsin excitation (figure 3*c,d*).

**Figure 4.**
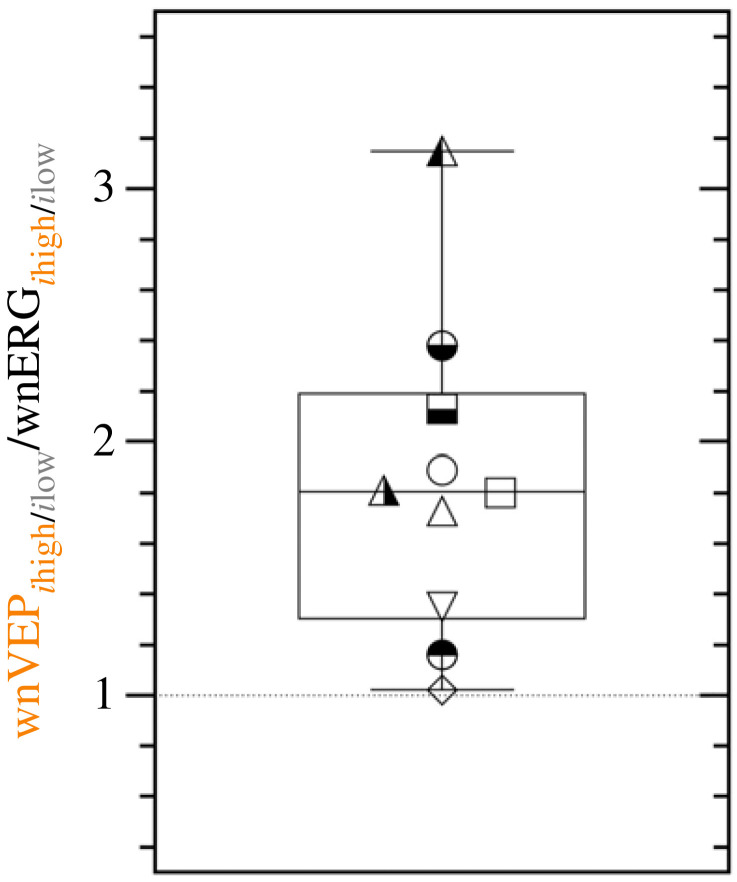
The wnVEP to wnERG amplitude ratio with higher (*i*_high_) to lower (*i*_low_) melanopsin excitation. Each observer is represented by a unique symbol (*n* = 10 observers). A ratio of < 1 indicates suppression and > 1 indicates amplification.

As a metric of the magnitude of the melanopsin-cone interaction, the melanopsin-mediated cone suppression and amplification was defined as the amplitude ratio (*μi*_high_/*μi*_low_) of the mean cone IRF amplitude for each observer. In the retina, the wnERG amplitude ratio was less than unity (N1P1 = 0.74 ± 0.08) indicating that melanopsin supresses the wnERG amplitude (figure 4). When measured in the cortex, the wnVEP amplitude ratio was greater than unity (N2P2 = 1.24 ± 0.07), indicating that melanopsin amplifies the wnVEP. The wnERG amplitude ratio was significantly different from the wnVEP ratio (*t*_9_ = 6.02, *p* = 0.0002). With reference to the suppressed retinal signals, the relative (total) amplification present in the cortex (*wnVEP*_N2P2 ratio_/*wnERG*_N1P1 ratio_) was therefore 1.84 ± 0.20. Ratios for implicit times were close to unity for both the wnERG (N1 = 1.01 ± 0.02; P1 = 0.99 ± 0.01) and wnVEP (N2 = 0.99 ± 0.01; P2 = 1.01 ± 0.00).

**Figure 5.**
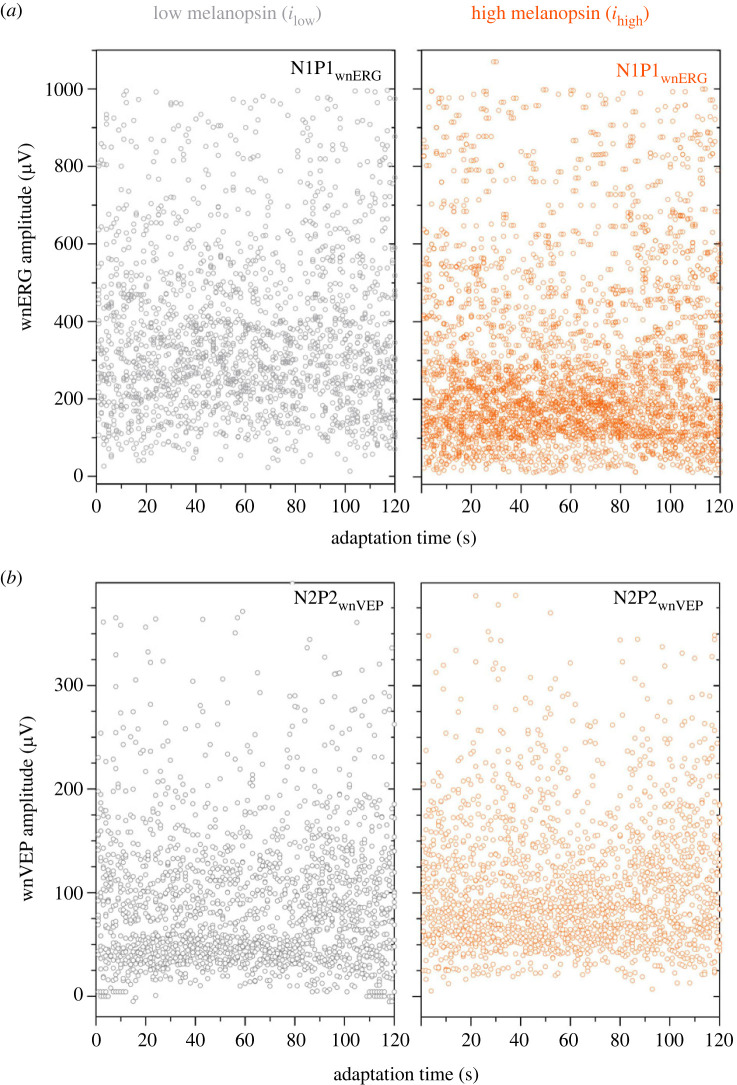
Short-term (2 min) stability of the cone-directed signals at the level of the retina (wnERG, upper panels) and primary visual cortex (wnVEP, lower panels). (*a*) The N1P1 amplitudes of the cone-directed white noise electroretinogram (wnERG) measured under low melanopsin (left panel, grey symbols; *i*_low_) or high melanopsin excitation (right panel, orange symbols; *i*_high_) plotted as a function of adaptation time for 10 observers (from a total of at least 1200 impulse response functions). (*b*) N2P2 amplitudes for the cone-directed white noise visual evoked potential (wnVEP).

To determine whether the melanopsin signal can adapt the cone ERG [11] and VEP on the short timescales measured here, the 120 IRFs derived from each 2 min wnERG and wnVEP recording sequence for each observer were analysed as a function of adaptation time (figure 5) using regression. The linear regressions were not significantly different from zero for any observer and so all recordings from the 10 observers (at least 1200 total recordings and typically 1900 recordings) were pooled to determine the global effect of melanopsin adaptation. The linear regressions were not significantly different from zero (N1 amplitudes: *i*_low_, *r*^2^ = 0.0009, *F*_1,1949_ = 1.92, *p* = 0.16; *i*_high_ N1, *r*^2^ = 0.06, *F*_1,2017_ = 3.3, *p* = 0.06; N1P1 amplitudes: *i*_low_, *r*^2^ = 0.0008, *F*_1,1949_ = 1.52, *p* = 0.21; *i*_high_, *r*^2^ = 0.001, *F*_1,2017_ = 2.92, *p* = 0.08), indicating that the IRFs of the wnERG did not significantly change during the short-term light adaptation (figure 5*a*). The wnVEP responses also did not significantly change during the 2 min light adaptation (N2P2 amplitudes: *i*_low_, *r*^2^ = 0.001, *F*_1,1895_ = 2.41, *p* = 0.12; *i*_high_, *r*^2^ = 0.001, *F*_1,1895_ = 1.74, *p* = 0.18) (figure 5*b*).

**Figure 6.**
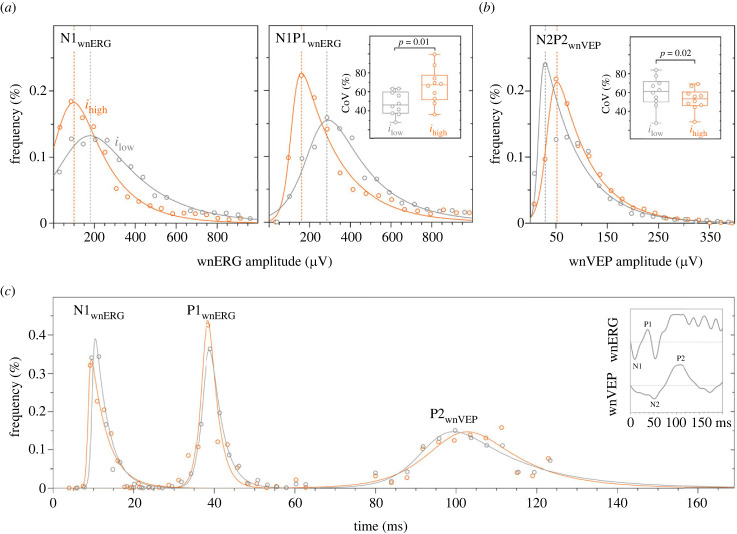
Effect of the biological efficacy of melanopsin on the amplitude (upper panels) and implicit time (lower panel) of cone signals measured at the level of the retina (wnERG) and primary visual cortex (wnVEP). (*a*) Frequency distributions of the cone-directed white noise electroretinogram (wnERG) measured under low melanopsin (*i*_low_; grey circles and lines) and high melanopsin excitation (*i*_high_; orange circles and lines). All observers (*n* = 10) and trials are analysed together (at least 1200 trials per condition). The solid lines are the best-fitting hyperbolic secant functions. Dotted vertical lines are the distribution modes. The inset (top middle panel) shows the intra-observer coefficient of variation (CoV) of the wnERG N1P1 amplitudes. (*b*) Visual evoked potential (wnVEP) amplitudes. The inset (top right panel) indicates the intra-observer coefficient of variation (CoV) of the wnVEP N2P2 amplitudes. (*c*) wnERG and VEP implicit times.

The absence of short-term retinal adaptation (figure 5) allowed us to determine the global effect of melanopsin on cone responses by plotting the frequency distributions of the retinal and cortical IRF data (pooled data from figure 5). The distributions were sampled in their optimal bin width and described using the best-fitting hyperbolic secant function because of their positive skew (figure 6). The melanopsin suppression of the cone-directed wnERG amplitude was evident as a leftward shift of the frequency distributions (figure 6*a*). The melanopsin enhancement of the cone-directed wnVEP was evident as a rightward shift of the frequency distribution (figure 6*b*). The implicit times became progressively longer with transmission from the outer retinal cone photoreceptors (N1 = ∼11 ms), bipolar cells (P1 = ∼39 ms) to the parieto-occipital regions of the visual cortex (P2 = ∼101 ms) (figure 6*c*), consistent with the expected event timings [58], but remained stable with the change in melanopsin excitation.

The signal to noise ratio of retinal ganglion cells is proportional to the luxotonic response of ipRGCs [59]. To determine whether the coding efficiency of the cone-pathway differs at a site prior to (i.e. the wnERG) and post-luxotonic ipRGC modulation of the cone signal (i.e. the wnVEP), we estimated the intra-observer variability across the 120 epochs for each observer by calculating the CoV for both amplitudes and implicit times (figure 6*a*,*b* insets). The CoV and signal to noise ratio are inversely related. The wnERG N1P1 amplitudes were significantly more variable with the higher melanopsin excitation (figure 6*a* inset; CoV: N1P1, *i*_low_ = 47.74 ± 3.90%, *i*_high_ = 66.20 ± 5.93%; *t*_9_ = 3.56, *p* = 0.01), whereas the pattern was opposite for the cortical evoked wnVEP responses (figure 6*b*, inset); the variability in N2P2 amplitudes significantly decreased with the higher (52.66 ± 3.68%) than lower melanopsin excitation (60.13 ± 5.13%) (*t*_9_ = 2.98, *p* = 0.02). To determine the co-variance between the melanopsin-mediated change in cone-directed wnERG or wnVEP amplitude and CoV as a measure of coding efficiency, we calculated the ratio of amplitude or CoV between the two melanopsin states. With increasing melanopsin excitation, the wnERG CoV increased 1.39-fold, mirroring the 1.37-fold corresponding decrease in the wnERG amplitude; also, the wnVEP CoV decreased by 1.12-fold, mirroring the 1.16-fold corresponding increase in the wnVEP amplitude. Intra-observer variability in the implicit times was invariant of the melanopsin excitation in the retina and brain as expected, given the similarity of the timings in the two conditions.

## 4. Discussion

By using photoreceptor silent substitution to precisely stabilize the rhodopsin activation under conditions of constant photometric luminance, the cortical evoked responses of the cone pathway were found to depend on the biological efficacy of the melanopsin excitation in the adapting light. A change in melanopsin excitation from low to high designed to capture elements of its total daytime variation modulated the amplitude of the cone-mediated visual pathway signal by 1.84× during transmission from the retina to the visual cortex. Cone signals that were initially supressed in the retina (N1P1 = −27%) were subsequently amplified when measured in the visual cortex (N2P2 = + 16%) with high melanopsin daylight conditions.

The suppression of the wnERG may signify action of melanopsin excitation-dependent gain control. In the retina, the detection of contrast over a wider dynamic range than the response of single cells depends on light adaptation to reduce the neuronal gain [60]. Because spatially antagonist centre-surround receptive fields only have indirect access to the ambient light level from luxotonic inputs via ipRGCs [1,61,62] and the graded output voltages from cones [63,64], the ipRGC signals may have a role in altering a cell's contrast response function by modulating gain within the inner retina, including between bipolar, amacrine and ganglion cells, which is a known location in primates for controlling the cone signal gain [65–68].

We observed that the cortical VEP is amplified with reference to the retinal ERG, and the amplitude dependence on the melanopsin excitation is reversed; what was attenuated in the outer retinal pathways represented in the ERG is amplified in the VEP. The adaptational response of the retina with the higher melanopsin excitation had a minor impact on the timing of the cortical evoked response, with the implicit times remaining stable during the short 2 min recording period at the level of retina and cortex (figure 5*c*). A relatively stable implicit time was not unexpected because melanopsin excitation suppresses the mouse flash ERG amplitude without affecting implicit time [40], and the b-wave implicit time does not change during 20 min adaptation [69]. In humans, the effect of melanopsin-dependent cone adaptation of the flash ERG manifests as faster (by 6–8 ms) b-wave implicit times only over longer time intervals from at least 15–120 min [11]. As determined from human behavioural estimates with photoreceptor directed light stimulation in reaction time [70], pupillometry [71,72], temporal summation [16] and subjective time expansion paradigms [73], in addition to cortical evoked potentials measured in melanopsin-only transgenic mice [74], the cone signals reach the brain before the melanopsin signal, in some conditions, by over 100 ms. The wnERG paradigm, which maintains the retina in a state of equilibrium more like natural viewing conditions than can a flash ERG, cannot temporally resolve these processes because the fast acting gain controls are operational within shorter timescales. For the flash ERG, the b-wave amplitudes are modulated by adaptation of the mass corneal potentials generated by cells in the inner nuclear layer [75,76], including feedback circuits from amacrines via interplexiform cells to horizontal cells [60,77,78], and such circuits are discussed later with reference to the melanopsin pathway. Taken together, melanopsin amplifies cortical responses without altering the signal transmission latency, indicating a role for melanopsin in optimizing the post-receptoral signal coding.

With increasing melanopsin excitation, the wnERG and VEP amplitudes exhibited an inverse covariance with variability. Cortical evoked responses have lower variability with higher melanopsin excitations (figure 6*b*, inset), indicative of increased signal to noise ratio for the same visual input, and therefore a subsequent increase in the amount of visual information that can be signalled to the brain. Similarly, recordings in mice reveal that the tonic firing rate of retinal ganglion cells is scaled to the ambient irradiance by the luxotonic response of ipRGCs to increase the number of spikes available to convey visual information in daylight [79] and, at the level of the dLGN, the signal to noise ratio of cone responses is increased [59]. The melanopsin-dependent changes in gain can also modify the functional response of the mouse M4 ipRGC subtype involved in pattern vision [80]. An analogous amplification method is used in power distribution grids in the built environment to increase signal transmission efficiency, wherein electrical power lines transmit high voltages at low currents to minimize power loss over long distances [81]. We infer that this resultant increase in information flow to the visual cortex with higher melanopsin states has the effect in humans of optimizing the post-receptoral cone signal coding of retinal outputs, and provides a physiological correlate of the melanopsin-driven enhancement of cone-mediated contrast sensitivity [15,16,19]. The Weber fraction for cone-mediated vision therefore decreases with higher melanopsin excitations at the same photometric luminance [18], indicating that visual detection only requires a smaller increment in the stimulus magnitude. Given that VEP contrast responses are highly correlated with visual contrast thresholds [82–85], an implication of our VEP findings for vision is that the Weber fraction can be regulated by melanopsin at a site distal to retinal bipolar cells, and within the visual cortex.

The melanopsin-dependent adaptation observed in the cone ERG can be localized to cells within the inner nuclear layer in primates [76]. Here, the gain could be regulated by the balance between retrograde excitatory glutamatergic amacrine cells inputs between ipRGCs and ganglion cells, and the anterograde inhibitory inputs via VGlut3 amacrine cells [86]. Although undefined in primates, this inhibitory pathway does involve dopaminergic amacrine cells in mice [13,87]. The extensive connectivity of ipRGCs across all levels of the mouse retina supports their capacity to modify visual signalling at multiple loci. In addition to their effect on photoreceptor [88] and bipolar cell function [40], the amplification may involve excitatory glutamatergic amacrine cell feedback from ipRGCs to ganglion cells [86,89], through ipRGC axon collaterals to the inner plexiform layer [90] and/or via gap junctions to other ganglion cells [91,92]. At least 15 brain areas receive direct ipRGC projections to mediate the effects of light on visual and non-visual functions [93]. In macaques, a higher order cortico-geniculate feedback pathway could amplify the signal via excitatory glutamatergic feedback from cortical layer VI to the LGN in as little as 37 ms [94]. The amplification could involve the excitatory convergence of different retino-geniculate inputs on single cortical neurones [95,96]. Our inference is that independent, luxotonic ipRGC inputs to the LGN and/or higher visual areas can regulate this excitatory effect on the non-melanopsin pathway signals, but this is yet to be directly tested *in vivo*.

To maximize the effect size of the melanopsin state on cone signalling while controlling the rhodopsin excitation, we evaluated the largest range in melanopsin states our system could produce. With this experimental approach, we observe that melanopsin directly affects cone signals present in the visual cortex. Based on previous work [10], we anticipate the suppressive effect of melanopsin on the cone-directed ERG will increase with both higher melanopsin excitations and adaptation levels, as will the melanopsin enhancement of the cone VEP. However, as is the case for human vision [97], the presence of nonlinearities at the extreme contrasts and light levels still need to be explored.

The relative sensitivities of cone, rod and melanopsin pathways are set by the spectral power distribution of the prevailing light. To understand how melanopsin-cone photoreceptor interactions optimize daylight contrast sensitivity, we spectrally engineered the light to modulate melanopsin independently of cones while stabilizing the rod activity. Our description of a functional amplification effect on cortical evoked visual responses with a change in the daylight melanopsin excitation provides an alternate view for regulating human visual contrast sensitivity. Spectrally tuning all five photoreceptor excitations [27] might itself provide a more practical experimental paradigm to evaluate time-of-day circadian effects systematically and efficiently in a laboratory setting, and to study afferent pathway modulation of higher order processes. It will be interesting to determine how the retinal and cortical melanopsin pathways modulate light-dependent effects on visual function and other electrophysiological signals, such as the electroencephalogram [93,98]. Although photometric luminance is the primary metric used in industry to specify the visual effectiveness of a light [99], we show that cone-mediated vision is also dependent on the ambient spectral and irradiance content of the light that drives the luxotonic melanopsin signal [38], independent of the photometric luminance and rod excitation, which is critical because melanopsin-rod-cone interactions have non-complementary effects on visual contrast sensitivity.

## Data Availability

Data are available from the Dryad Digital Repository [100].
